# CT assessment of total abdominal muscle area index (TAMAI) as a predictive tool for early post operative complications in laparoscopic sleeve gastrectomy: a prospective case-control study

**DOI:** 10.1186/s12876-025-04176-4

**Published:** 2025-08-05

**Authors:** Islam Haney Shawali, Yara ELhefnawi, Mohammed ElShwadfy Nageeb, Bahaa Eldin Mahmoud

**Affiliations:** 1 Diagnostic and interventional radiology, Faculty of medicine, Cairo University, Cairo, Egypt; 2 Faculty of medicine, Cairo University, Cairo, Egypt

**Keywords:** Sarcopenia, Obesity, Metabolic bariatric surgery, Postoperative complications

## Abstract

**Background:**

In metabolic bariatric surgery (MBS) a lot of focus is made on preoperative risk assessment to enhance patient’s baseline performance and improve postoperative clinical outcomes. The aim of this study is to assess pre-operative sarcopenia by computed tomography (CT) scan, as a predictive tool for early post-operative complications in candidates for MBS.

**Methods:**

This is a single center prospective case-control study. The study includes using non-contrast CT cuts at L3 vertebra level to measure total abdominal muscle area (TAMA) and visceral fat area (VFA). TAMA was indexed to the patient height and VFA/TAMAI ratio was estimated. Models for predicting postoperative complications were made for TAMA alone, TAMAI alone, VFA alone and VFA/TAMAI ratio to assess each factor’s reliability in predicting postoperative complications.

**Results:**

The study enrolled 30 patients who underwent laparoscopic sleeve gastrectomy (LSG); 14 experience early post-operative complications in the cases arm, matched against 16 in the control arm. TAMA and TAMAI showed a significant association with early post-operative complications.

**Conclusion:**

Our findings suggest that TAMA and TAMAI, measured by non-contrast CT as markers for sarcopenia, may be associated with early post-operative complications for laparoscopic sleeve gastrectomy (LSG) patients.

## Introduction

The obesity crisis is a worldwide pandemic. Its metabolic consequences link it directly with various health risks. It also poses a great economic burden on health care worldwide [1]. Metabolic bariatric surgery (MBS) offered a new frontier to obesity management. Achieving relatively rapid weight loss outcomes, laparoscopic MBS have become more utilized in healthcare [2]. Despite their low-risk minimally invasive nature, complications are still a recognized risk [3].

To minimalize inadvertent outcomes, preoperative risk assessment models have been designed to allocate and modify any preoperative risk factor associated with inadvertent outcome, thus creating optimum baseline preoperative health status [4].

A range of preoperative predictive factors for postoperative complications in MBS have been identified in the literature. These include patient-related factors such as age, sex, body mass index (BMI), American Society of Anesthesiologists (ASA) score, smoking status, and the presence of comorbidities including type 2 diabetes, hypertension, obstructive sleep apnea, and cardiovascular disease [4, 5]. Laboratory markers such as low serum albumin and elevated CRP have also been linked to adverse surgical outcomes [6]. In addition, functional status and frailty—assessed via mobility tests or comprehensive geriatric evaluation—have shown predictive value, especially in older adults [7].

Beyond clinical factors, recent research has emphasized the role of body composition as an important predictor. Specifically, sarcopenia (low muscle mass) and sarcopenic obesity have been associated with increased risk of complications, delayed recovery, and impaired wound healing following MBS [8, 9]. Visceral fat accumulation, rather than total fat mass alone, has been independently associated with higher rates of staple line leak and cardiopulmonary events [10].

Several risk stratification models have been proposed—such as the Obesity Surgery Mortality Risk Score (OS-MRS), the Bariatric Surgery Index for Complications (BASIC), and the Edmonton Obesity Staging System (EOSS)—all of which integrate a combination of the above variables to estimate perioperative risk [11–13]. However, these models often lack integration of quantitative imaging biomarkers, which may provide added predictive power.

A 2017 systematic review by Jones et al. highlighted that radiologically determined sarcopenia is a significant predictor of both morbidity and mortality in abdominal surgery [14]. Similarly, a meta-analysis by Nuijten et al. (2022) showed that lean body mass loss is an early and significant postoperative event in MBS patients, emphasizing the importance of its preoperative evaluation [15].

The integration of cross-sectional imaging, particularly CT, into preoperative assessment offers a non-invasive and reproducible method to evaluate skeletal muscle and fat compartments. This concept, often referred to as “opportunistic imaging,” allows clinicians to derive prognostic biomarkers such as total abdominal muscle area (TAMA) and visceral fat area (VFA) from routine CT scans [16]. Identifying high-risk patients using these imaging-based indices could guide tailored prehabilitation strategies, such as nutrition optimization and resistance training, to reduce complication rates and improve outcomes.

Our aim is to explore CT assessment of sarcopenia as one of the potential prognostication tools for complications post-laparoscopic sleeve gastrectomy (LSG).

## Materials and methods

### Study design and patients

We conducted a single-center prospective case-control study design. Cases were picked over the period of 12 months, from the period of January 2023 to December 2023. Controls from the same period were then matched to cases. Selection was carried out from Cairo University Hospital database.

*Inclusion criteria* involved (1) patient who underwent LSG for treatment of obesity, (2) patients older than 18 years of age, and (3) cases defined as patients who developed early postoperative complications that occurred within 30 days of LSG. Complications were defined as per the Clavien-Dindo classification of surgical complications. Only complications of degree II or higher were considered; this includes but is not limited to leakage, bleeding, serious surgical site infections, cardiopulmonary compromise, or requiring readmission [8].

*Exclusion criteria* included patients undergoing revision MBS or other surgeries involving anterior abdominal muscle wall like hernia repairs.

### Demographics, anthropometry data and surgical technique

Clinical history and demographics were collected for the general surgery department database. This includes weight, height and BMI measured as part of preoperative routine assessment. Hypertension was defined as systolic blood pressure > 140 mmHg or diastolic blood pressure > 90 mmHg. Diabetes mellitus was defined as per the NIH diagnostic criteria. Cardiac conditions included previous diagnosis of ischemic heart disease or acute coronary syndrome, chronic valve disease that requires medication, or heart failure.

All patients underwent a standardized LSG procedure performed under general anesthesia. Pneumo-peritoneum was established using a Veress needle or open (Hasson) technique, followed by placement of five trocars in a standard configuration. The greater curvature of the stomach was mobilized, starting approximately 4–6 cm proximal to the pylorus and extending up to the angle of His, with complete dissection of the short gastric vessels. A 36 French bougie was inserted along the lesser curvature to calibrate the sleeve. Gastric transection was performed using a linear stapler, starting 4 cm from the pylorus and proceeding vertically toward the fundus. Staple line reinforcement with running sutures or absorbable buttressing material was used selectively. Intraoperative leak testing was performed using air insufflation or methylene blue in most cases. All procedures were conducted by two senior bariatric surgeons, each with over 10 years of operative experience in advanced laparoscopic and metabolic surgery, ensuring uniform technique and minimizing inter-operator variability.

### CT scan acquisition

Since CT scans of the abdomen is not part of the routine preoperative assessment at our center, a collaboration with general surgery department was done to perform preoperative non-contrast CT scan to enlisted LSG patients to evaluate baseline body composition. The purpose of imaging was solely to assess the prognostic value of sarcopenia and visceral fat for predicting early postoperative complications, and not to monitor postoperative changes in visceral fat or muscle mass.

Written consent was taken from all subjects. CT studies were done on GE revolution Evo 128 slice.

### Measurements

All CT studies were processed on PaxeraUltima PACS. Total abdominal muscle area (TAMA) was calculated at the level of L3 vertebral body. Total visible muscle cross sectional area -including the anterior abdominal wall muscles, paraspinal muscles and psoas muscle- was manually traced by a trained radiologist to calculate the area in cm^2^. Two tracings were made by trained radiologist and the estimated average was taken. TAMA was corrected to the patient’s height squared calculating TAMA index (TAMAI) (m^2^/cm^2^).

**Fig. 1:**
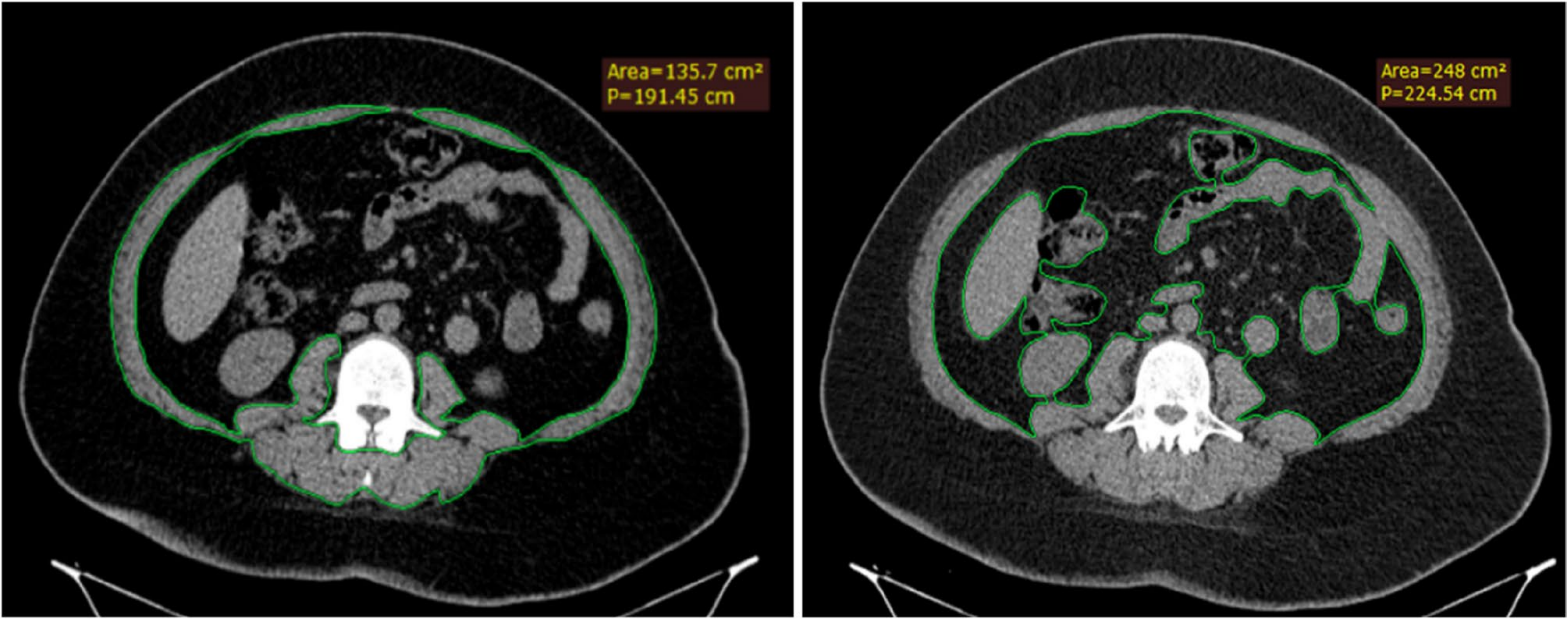
Preoperative non-contrast CT scan from the control arm; TAMA and VFA calculated by manually tracing the total visible abdominal muscles at L3 vertebra as shown. **a** TAMA = 135.7 cm^2^** b** VFA = 248 cm^2^. Given the patient’s height of 1.58 m, TAMAI was estimated 54.4 cm^2^/m^2^ and VFA/TAMAI ratio 4.6

**Fig. 2:**
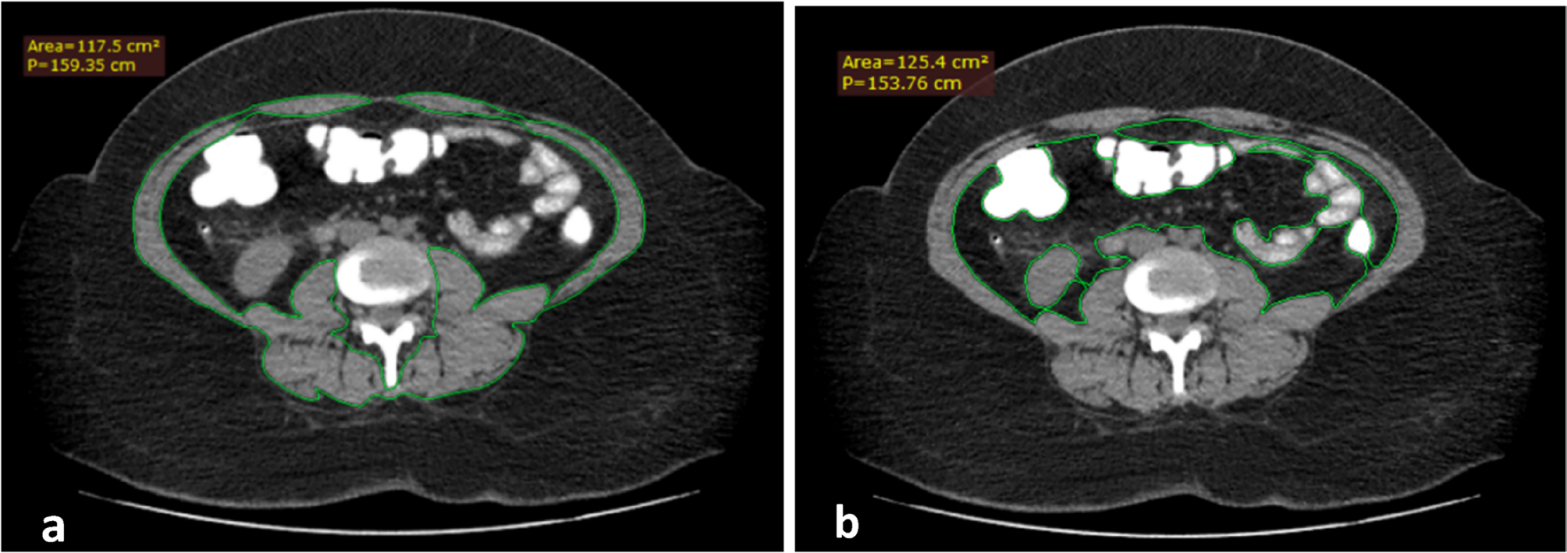
37 years old from the cases group, BMI 41.7 kg/m^2^, weight 117 kg, height 1.68 m. CT axial cut at L3 shows **a** TAMA = 117.5 cm^2^, **b** VFA = 125.4 cm^2^, TAMAI 42 cm^2^/m^2^, VFA/TAMAI ratio 3. This patient had postoperative leakage managed by megastent with later recovery

Visceral fat at the same level was also manually traced to calculate its cross-sectional area in m^2^, defining visceral fat area (VFA). The average time spent by the radiologist in measuring both TAMA and VFA is 4 min 15 s (Figs. 1 and 2).

### Statistical analysis

Statistical analysis was conducted using SPSS 27th edition. Numeric variables were presented in mean, standard deviation, minimum and maximum. It was compared between groups using Mann Whitney U test, normality testing was conducted using Shapiro Wilk test.

Categorical variables were presented in count and percentage, it was compared between groups using Pearson Chi^2^ test. Sensitivity analysis was conducted to estimate the cutoff value for prediction of postoperative complications, along with its sensitivity, specificity, and accuracy. P values < 0.05 was considered significant.

Models for predicting postoperative complications were made for TAMA alone, TAMAI alone, VFA alone and VFA/TAMAI ratio to assess each factor’s reliability in predicting postoperative complications. Receiver operating characteristic (ROC) analysis was performed, and the area under the curve (AUC) was calculated for each of the models to compare their predictive value.

## Results

### Participants characteristics

We enrolled 30 patients who underwent LSG, 14 cases (complicated) were matched against 16 controls with no complications, making a total of 30 cases.

**Fig. 3:**
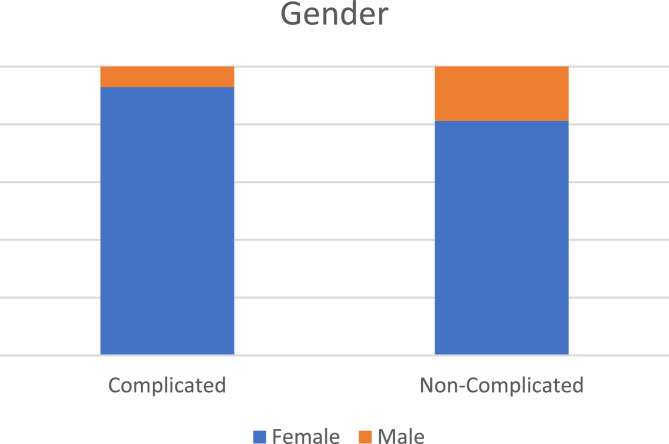
Bar chart showing gender distribution among study groups

Patients showed a mean age of 36.6 ± 10.9 years, females accounted for the majority of the included patients (Fig. 3). Comparison of age, gender, BMI, weight, and height between the two arms showed no statistical significance with *p* values > 0.05 (Table 1). Smoking, previous surgeries, hypertension, diabetes, and cardiac diseases showed no statistically significant differences between study two arms with *p* value > 0.05.

**Table 1:** Comparison of smoking, previous surgeries, and comorbidities among study groups

		Complicated		Uncomplicated		
		Count	%	Count	%	*P* value
Sex	Female	13	92.9%	13	81.3%	0.110
	Male	1	7.1%	3	18.8%	
Smoking	No	13	92.9%	15	93.8%	0.922
	Yes	1	7.1%	1	6.3%	
Previous surgery	No	7	50.0%	9	56.3%	0.732
	Yes	7	50.0%	7	43.8%	
Hypertension	No	11	78.6%	11	68.8%	0.544
	Yes	3	21.4%	5	31.3%	
Diabetes	No	10	71.4%	13	81.3%	0.526
	Yes	4	28.6%	3	18.8%	
Cardiac	No	13	92.9%	16	100.0%	0.277
	Yes	1	7.1%	0	0.0%	
Other	Fatty liver	0	0.0%	1	6.3%	0.392
	Hyperlipidemia	0	0.0%	1	6.3%	
	No	14	100.0%	14	87.5%	
		Mean ± SD	Min-Max	Mean ± SD	Min-Max	
Age		36.1 ± 10.8	21–55	36.9 ± 11.3	16–53	0.822
BMI (kg/m^2^)		47 ± 8.3	32–64	48.4 ± 7.9	40.4–70.8	0.951
Weight (kg)		127.4 ± 27.7	90–175	127.9 ± 19.2	85–170	0.637
Height (m)		1.6 ± 0.1	1.5–1.8	1.6 ± 0.1	1.5–1.8	0.984

### Post-operative complications

**Fig. 4:**
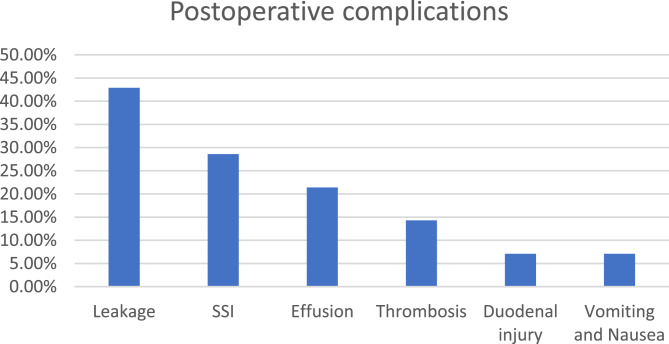
Bar chart showing distribution of postoperative complications

Leakage was the most common postoperative complication accounting for 42.9% of the cases arm, followed by SSI in 28.6%, pleural effusion 21.4%, thrombosis in 14.3%, duodenal injury in one patient and vomiting in one patient (Fig. 4).

### Comparison of body composition between cases and controls

**Fig. 5:**
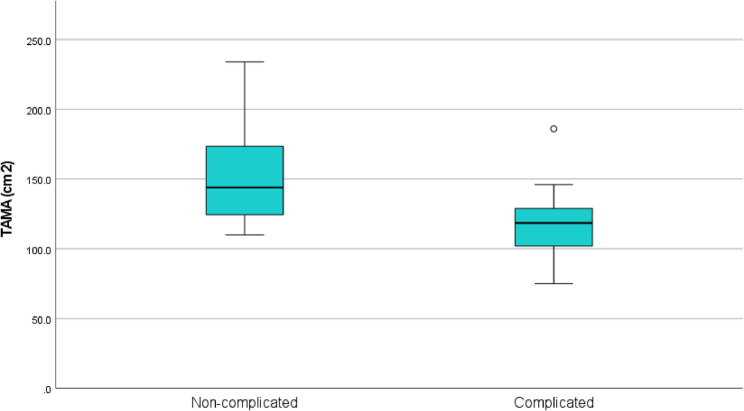
Box plot showing TAMA distribution among study groups

**Fig. 6:**
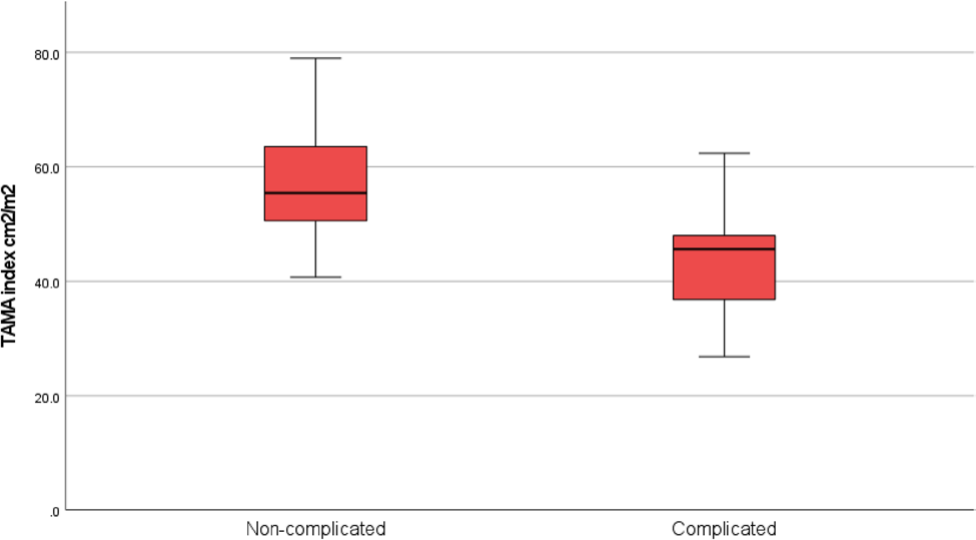
Box plot showing TAMAI distribution among study groups

TAMA and TAMAI were significantly lower among complicated cases compared to controls; mean TAMA 117.8 ± 28.6 cm^2^ versus 152.3 ± 36.5 cm^2^ with p value 0.004. Mean TAMAI 45 ± 9.7 versus 57.2 ± 10.2, cm^2^/m^2^ with *p* value 0.002. However, VFA and VFA/TAMAI ratio showed no statistically significant differences between groups with p values 0.854, and 0.064 respectively (Table 2) (Figs. 5 and 6).

**Table 2:** Comparison of TAMA, TAMAI, VFA, and VFA/TAMAI ratio between study groups

	Group				
	Complicated		Uncomplicated		
	Mean ± SD	Min-Max	Mean ± SD	Min-Max	*P* value
TAMA (cm^2^)	117.8 ± 28.6	75–186	152.3 ± 36.5	110–234	0.004
TAMAI (cm^2^/m^2^)	45 ± 9.7	26.8–62.4	57.2 ± 10.2	40.7–79	0.002
VFA (cm^2^)	189 ± 74	110–334	187 ± 72	81–332	0.854
VFA/TAMAI ratio	4.3 ± 1.7	2-7.4	3.2 ± 1	1.7–4.7	0.064

Logistic regression model showed that patients with TAMA < 122 cm^2^ are 7.8 times more likely to suffer postoperative complications with 95% CI 1.5–41.2 and *p* value 0.016. TAMAI < 48 cm^2^/m^2^ showed an odds ratio of 7.5 (95% CI 1.5–37.9) for development of postoperative complications with *p* value 0.015 (Table 3).

**Table 3:** Univariate logistic regression model showing TAMA and TAMAI as predictors for postoperative complications

	B	*P* value	OR	95% CI
TAMA (< 122cm^2^)	2.054	0.016	7.8	1.5–41.2
TAMAI (< 48 cm^2^/m^2^)	2.015	0.015	7.5	1.5–37.9

### Predictive models for postoperative complications

Receiver operating characteristic (ROC) analysis was performed, and the area under the curve (AUC) was calculated for each of the models to compare their predictive value.

TAMA can significantly predict postoperative complications with patients who have < 122 cm^2^, with p value 0.006, sensitivity 81.3%, specificity 65%, and AUC 79.7%. Similarly, TAMAI using a cutoff value < 48 cm^2^/m^2^, with sensitivity 81.3%, specificity 79%, AUC 82.1% and *p* value 0.003.

**Fig. 7:**
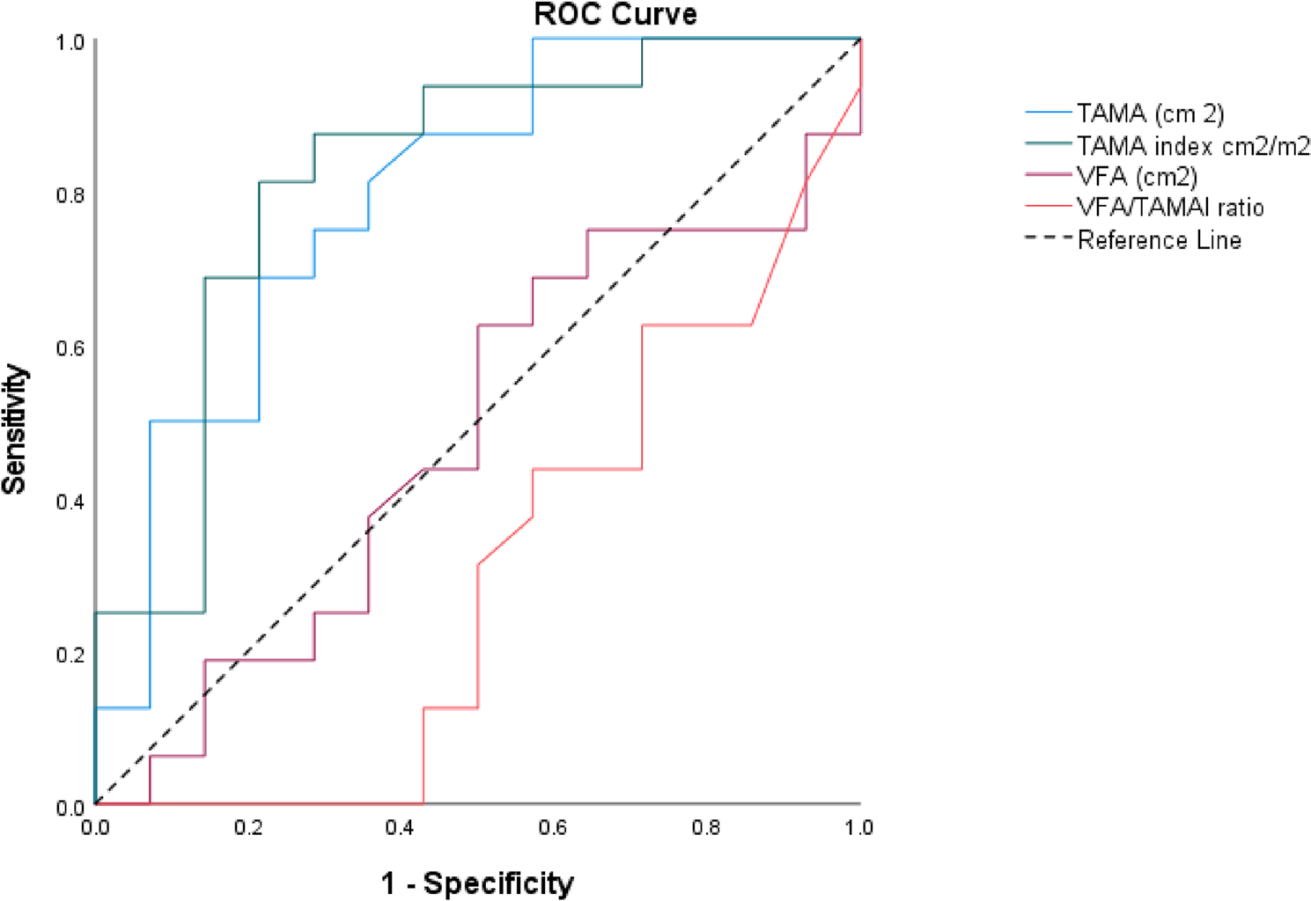
ROC curve showing predictability of TAMA, TAMAI, VFA and VFA/TAMAI ratio for postoperative complications

However, VFA and VFA/TAMAI ratio showed no statistically significant predictability for postoperative complications with p values 0.852, and 0.061 respectively (Table 4 and Fig. 7). 

**Table 4:** Sensitivity analysis showed the predictability of TAMA, TAMAI, VFA and VFA/TAMAI ratio for postoperative complications

	AUC	*P* value	95% CI	Cutoff	Sensitivity	Specificity
**TAMA (cm** ^**2**^ **)**	0.797	0.006	0.633–0.96	122	81.3%	65%
**TAMAI cm** ^**2**^ **/m** ^**2**^	0.821	0.003	0.663–0.98	48	81.3%	79%
**VFA (cm** ^**2**^ **)**	0.48	0.852	0.267–0.693			
**VFA/TAMAI ratio**	0.299	0.061	0.106–0.492			

## Discussion

Assessing the correlation between sarcopenia and postoperative clinical outcome is valuable because sarcopenia is a potentially modifiable risk factor [9]. Several studies suggested that adjusting perioperative nutritional plan and exercising helped improve outcome. Aoyama et al. in a review article coined multiple clinical trials studying the effect of pre-operative rehabilitation program on the clinical outcome of gastric cancer patients with sarcopenia undergoing gastrectomy. Preoperative rehabilitation programs targeting sarcopenia showed clinical benefits for these patients [10].

Sarcopenic obesity (SO) is now a commonly recognized phenotype in obesity, many of MBS patients will fall in this category. A 2024 study by González Arnáiz et al., showed a SO prevalence of 13–23% among 124 MBS candidates using DEXA, BIA, and handgrip strength for assessment [17]. Not only does sarcopenic obesity reflect on recovery and clinical outcome, sarcopenia may even worsen after MBS. This is especially true since an element of high protein loss is anticipated during the heavy weight loss phase postoperatively, where the already low lean body mass (LBM) drops further. A cohort study by Alba et al. showed that even though physical function improves after weight loss, lean body mass, and absolute handgrip strength drops in the setting of rapid weight loss [11, 12].

A systematic review and meta-analysis done by Nuijten and colleagues concluded that LBM, fat-free mass, and skeletal muscle mass were predominantly lost within 3-month post-MBS, and they highlighted the importance of implementing perioperative measures to mitigate this [13].

**Fig. 8:**
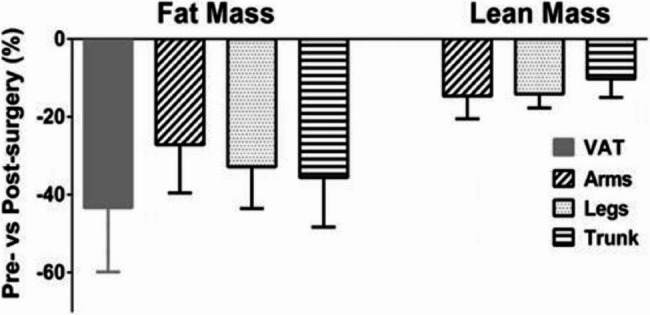
Regional percentage drop in fat mass and lean mass 6 months after MBS by Zhou et al. [14]

Figure 8 from a study by Zhou et al., shows the relative drop in LBM as well as fat mass 6 months after MBS, about 12% lean mass is lost. This study suggests that the quantitative loss of muscle mass overrides the positive qualitative metabolic effect of weight loss/fat mass loss [14].

To counteract this decrease in LBM and protein loss, most MBS protocols utilize a detailed nutritional and lifestyle management plan perioperatively to prevent short and long-term morbidity. During the International Federation for the Surgery of Obesity and Metabolic Disorders (IFSO) 2023 consensus conference, *VM. Arcone* coins the importance of nutritional planning and high protein intake to prevent short and long-term morbidity. Experts agreed that performing diagnostic studies to identify sarcopenia is advisable in at-risk individuals both before and after MBS [15].

Malnutrition risk assessment tools are also being explored in surgical setting to help optimize postoperative outcome. Petra et al., investigated various nutritional screening tools for different surgical settings. The combination use of nutrition tools with TAMAI could further optimize the preoperative status of the patient [18]. 

Despite that, routine preoperative assessment of sarcopenia in MBS patients is yet to be proven valuable. Preoperative detection of individuals with low LBM would provide an opportunity for more targeted therapy and hence optimizing patient’s preoperative status and improving outcome.

Our study showed that body composition can be used as a predictive tool for early post-operative complications. Cross sectional total abdominal muscle area (TAMA) indexed to patient’s height (TAMA index/TAMAI) showed significant association with higher complication rate. Our statistical analysis showed the possibility of using a cut-off value for TAMAI to predict the likelihood of postoperative complications; with a cutoff value < 48 cm^2^/m^2^, with sensitivity 81.3%, specificity 79%, AUC 82.1% and p value 0.003.

Leakage was the most encountered complication in our study. In LSG, leakage is one of the most feared complications, increasing length of hospital stay and morbidity. This coins the importance of establishing proper predictive model for this complication [19]. 

CT scan is one of the best modalities to assessing sarcopenia. It has the advantage of providing quantitative data on muscle mass and composition (including inter and intramuscular fat). Although no international standardization is set for measurement of body composition using CT, the two most common approaches are to measure all visualized muscles at the L3 level or measure just the psoas muscles at the L4 level. Measuring paraspinous muscles at D12 is also described in studies considering chest diseases [16]. Our study measured all visualized skeletal muscle area at L3 vertebral body.

Although preoperative CT is not a routine bariatric investigation, implementing it in our study proved easy access, cost effectiveness and with only minimal risk of radiation exposure with no iodinated contrast exposure risk.

Only a few studies tested the correlation between sarcopenia and MBS. Xin Yu et al. in their case control study shows a positive association between VFA/TAMAI ratio and postoperative complications. However, they did not find a significant correlation between TAMAI alone and complications, which is contradicting to our study results [20]. Another study by Martin Galliard and colleagues showed a positive correlation between CT assessed sarcopenia and postoperative gastric leak in bariatric patients [21].

Pre-operative assessment of sarcopenia has also been studied in various other surgical intervention. Nauheim and colleagues showed positive correlation between sarcopenia and increased length of hospital stay and postoperative complications in pancreatic cancer patients undergoing pancreaticoduodenectomy [22].

Sarcopenia has also been associated with and considered an independent prognostic factor for mortality after hepatectomy for hepatocellular carcinoma, after non-small‐cell lung cancer surgery, and after descending thoracic aortic aneurysm repair surgeries. All these studies used CT as the assessment tool for muscle index [23–25].

Sarcopenia is also associated with poor mobility and increased risk of fall. This would be exacerbated in the postoperative settings since it is associated with generalized weakness, disorientation, and delirium especially in the elderly [26, 27].

Some limitations in our study however should be discussed. Firstly, our sample size was very limited. Secondly, males were greatly underrepresented in our study and cut off values did not consider each gender separately, which might induce bias since all studies consider sex-specific cutoff values for the diagnosis of sarcopenia. Our CT also had weight limit of about 180 kg, this means part of MBS candidates were excluded from our study.

Despite showing association with postoperative complications, none of our patients fulfilled the agreed upon TAMAI cutoff for sarcopenia diagnosis in females, which is usually 2 standard deviations below muscle area of average healthy adult; that is at the level of L3 range from SMI < 40.2–52.4 cm^2^/m^2^ in men and SMI < 31.6–38.5 cm^2^/m^2^ in women according to the most recent publications in 2020. A reason for that might be (1) our measurement method which involved measuring the total visible muscle area in the ROI without a specified attenuation density (attenuation range of − 29 to + 150 HU is usually used). This means intramuscular fat was considered too in our measurement [28]. (2) Both men and women were studied as one group regarding TAMA and outcomes, the naturally higher TAMA in men tilted the values towards the relatively higher values in the male population. (3) Lack of national consensus of the normal skeletal muscle mass area in our population [29, 30].

Cut-off values of sarcopenia and sarcopenic obesity (SO) are yet to be standardized for each modality. For example, a study by Bufano et al. in 2024 calculated new cut-off values for sarcopenia in morbidly obese patients calculated by BIA [31].

Another limitation in our study is the lack of functional assessment of sarcopenia. All sarcopenia consensus groups recommend using both muscle quantitative data and clinical assessment of muscle function in the diagnosis of sarcopenia [32]. However, functional assessment tools show greater variability than muscle mass measurement, and some experts advocated for removing excluding it from the diagnosis of sarcopenia [16].

## Conclusion

Our findings suggest that TAMA and TAMAI, measured by non-contrast CT as a marker for sarcopenia, may be associated with early post-operative complications for LSG patients. While preliminary, these results indicate that TAMA and TAMAI could have a role in preoperative risk assessment of LSG and might help guide preoperative interventions aimed at reducing complication risk and improving postoperative outcomes.

## Data Availability

Data generated or analysed during this study are included in this published article, however the raw data are available from the corresponding author on reasonable request.

## Abbreviations

CT: Computed Tomography
MBS: Metabolic bariatric surgery
LSG: Laparoscopic sleeve gastrectomy
TAMA: Total abdominal muscle area
TAMAI: Total abdominal muscle area index
VFA: Visceral fat area
SO: Sarcopenic obesity
IGF-1: Insulin derived growth factor 1
DAG: Diacylglycerol
BIA: Bioelectrical Impedance Analysis
DXA: Dual energy X-ray absorptiometry
BMI: Body mass index
LBM: Lean body mass
AUC: Area under curve
ROI: Region of interest
EWSGSOP: European Working Group on Sarcopenia in Older People
IWGS: International Working Group on Sarcopenia
IFSO: International Federation for the Surgery of Obesity and Metabolic Disorders

## References

[1] -Wondmkun YT. Obesity, insulin resistance, and type 2 diabetes: associations and therapeutic implications. Diabetes Metabolic Syndrome Obesity: Targets Therapy. 2020;13:3611–6. 10.2147/DMSO.S275898. 33116712 10.2147/DMSO.S275898 PMC7553667

[2] -Angrisani L, Santonicola A, Iovino P, Formisano G, Buchwald H, Scopinaro N. Bariatric surgery worldwide 2013. Obes Surg. 2015;25(10):1822–32. 10.1007/s11695-015-1657-z. 25835983 10.1007/s11695-015-1657-z

[3] Arterburn DE, Telem DA, Kushner RF, Courcoulas AP. Benefits and risks of bariatric surgery in adults: a review. JAMA. 2020;324(9):879–87. 10.1001/jama.2020.12567. 32870301 10.1001/jama.2020.12567

[4] -Sall AR, Jones MW. Bariatric surgery preoperative assessment. [Updated 2023 Jul 8]. In: StatPearls [Internet]. Treasure [internet].land (FL): StatPearls Publishing; https://www.ncbi.nlm.nih.gov/books/NBK594256/37603647

[5] Coblijn UK, Karres J, de Raaff CAL, et al. Predicting postoperative complications after bariatric surgery: the bariatric surgery index for complications, BASIC. Surg Endosc. 2017;31:4438–45. 10.1007/s00464-017-5494-0. 28364156 10.1007/s00464-017-5494-0 PMC5666042

[6] -Halim I, Koak Y. Preoperative risk scoring systems in bariatric surgery. In: Agrawal S, editor. Obesity, bariatric and metabolic surgery. Cham: Springer; 2016. 10.1007/978-3-319-04343-2_14.

[7] Pinotti E, Montuori M, Borrelli V, et al. Sarcopenia: what a surgeon should know. Obes Surg. 2020;30:2015–20. 10.1007/s11695-020-04516-1. 32124217 10.1007/s11695-020-04516-1

[8] -Dindo D. The Clavien–Dindo classification of surgical complications. In: Cuesta M, Bonjer H, editors. Treatment of postoperative complications after digestive surgery. London: Springer; 2014. 10.1007/978-1-4471-4354-3_3.

[9] -Samuel C. Sarcopenia: Causes, Consequences, Prevention and Treatment. The Singapore family physician. 2018;44(5): 11. SFP_Cover_102018 (cfps.org.sg).

[10] -Aoyama T, Nakazono M, Nagasawa S, Segami K. Clinical impact of a perioperative exercise program for sarcopenia and overweight/obesity gastric cancer. In Vivo (Athens Greece). 2021;35(2):707–12. 10.21873/invivo.12311. 33622863 10.21873/invivo.12311 PMC8045110

[11] Alba DL, Wu L, Cawthon PM, Mulligan K, Lang T, Patel S, King NJ, Carter JT, Rogers SJ, Posselt AM, Stewart L, Shoback DM, Schafer AL. Changes in lean mass, absolute and relative muscle strength, and physical performance after gastric bypass surgery. J Clin Endocrinol Metab. 2019;104(3):711–20. 10.1210/jc.2018-00952. 30657952 10.1210/jc.2018-00952 PMC6339456

[12] Jones K, Gordon-Weeks A, Coleman C, et al. Radiologically determined sarcopenia predicts morbidity and mortality following abdominal surgery: a systematic review and meta-analysis. World J Surg. 2017;41:2266–79. 10.1007/s00268-017-3999-2. 28386715 10.1007/s00268-017-3999-2 PMC5544798

[13] -Nuijten MAH, Eijsvogels TMH, Monpellier VM, Janssen IMC, Hazebroek EJ, Hopman MTE. The magnitude and progress of lean body mass, fat-free mass, and skeletal muscle mass loss following bariatric surgery: A systematic review and meta-analysis. Obes Reviews: Official J Int Association Study Obes. 2022;23(1):e13370. 10.1111/obr.13370. 10.1111/obr.13370 PMC9285034 34664391

[14] Scoubeau -NaZC, Forton K, Loi P, Closset J, Deboeck G, Moraine J-J, Klass M, Vitalie Faoro.; (2022) Lean Mass Loss and Altered Muscular Aerobic Capacity after Bariatric Surgery. Obes Facts. 2022; 15(2): 248–256. 10.1159/000521242 10.1159/000521242 PMC9021623 35086094

[15] -International Federation for the Surgery of Obesity and Metabolic Disorders (IFSO). Consensus on definitions and clinical practice guidelines for patients considering metabolic-bariatric surgery. ISBN: 9788894466645, International Federation for the Surgery of Obesity and Metabolic Disorders (ifso.com). 2023.

[16] -Leon Lenchik Robert D, BoutinSemin. Sarcopenia: Beyond Muscle Atrophy and into the New Frontiers of Opportunistic Imaging, Precision Medicine, and Machine Learning Musculoskelet Radiol 2018;22(03): 307–322 10.1055/s-0038-1641573 10.1055/s-0038-1641573 29791959

[17] Petra -Georgia, Kritsotakis EI, Gouvas N, Schizas D, Toutouzas K, Karanikas M, Pappas-Gogos G, Stylianidis G, Zacharioudakis G, Laliotis A, Christodoulidis G, Kehagias I, Konstantinos Lasithiotakis. The MATS study group multicentre prospective study on the diagnostic and prognostic validity of malnutrition assessment tools in surgery. Br J Surg. 2025;112(2):znaf013. 10.1093/bjs/znaf013. 40037524 10.1093/bjs/znaf013 PMC11879291

[18] -Verras G-I, Mulita F, Lampropoulos C, Kehagias D, Curwen O, Antzoulas A, Panagiotopoulos I, Leivaditis V, Kehagias I. Risk factors and management approaches for staple line leaks following sleeve gastrectomy: A Single-Center retrospective study of 402 patients. J Personalized Med. 2023;13(9):1422. 10.3390/jpm13091422. 10.3390/jpm13091422 PMC10532722 37763189

[19] -González Arnáiz E, Ariadel Cobo D, Estébanez B, Galindo B, Pintor D, de la Maza B, Urioste Fondo A, Dameto Pons C, Cuevas MJ, Ballesteros Pomar MD. Prevalence of sarcopenic obesity according to different diagnostic methods and cut-off points in candidates for bariatric surgery. Clin Nutr. 2024;43(5):1087–93. 10.1016/j.clnu.2024.03.015. 10.1016/j.clnu.2024.03.015 38579371

[20] Yu X, Huang YH, Feng YZ, Cheng ZY, Wang CC, Cai XR. Association of body composition with postoperative complications after laparoscopic sleeve gastrectomy and Roux-en-Y gastric bypass. Eur J Radiol. 2023;162: 110768. 10.1016/j.ejrad.2023.110768. 36913816 10.1016/j.ejrad.2023.110768

[21] Gaillard M, Tranchart H, Maitre S, et al. Preoperative detection of sarcopenic obesity helps to predict the occurrence of gastric leak after sleeve gastrectomy. Obes Surg. 2018;28:2379–85. 10.1007/s11695-018-3169-0. 29500672 10.1007/s11695-018-3169-0

[22] Nauheim DO, Hackbart H, Papai E, Moskal D, Yeo CJ, Lavu H, Nevler A. Preoperative sarcopenia is a negative predictor for enhanced postoperative recovery after pancreaticoduodenectomy. Langenbecks Arch Surg. 2022;407(6):2355–62. 10.1007/s00423-022-02558-w. 35593934 10.1007/s00423-022-02558-w

[23] Voron -Thibault, Lambros T, Daniel P, Frederic P, Alexis L, Philippe C, Chady S, Alain L, Azoulay Daniel. (2015) Sarcopenia Impacts on Short- and Long-term Results of Hepatectomy for Hepatocellular Carcinoma. Ann Surg. 2015;261(6):1173–1183.| 10.1097/SLA.0000000000000743 10.1097/SLA.0000000000000743 24950264

[24] -Tanaka A, Sandhu HK, Rstum A, Afifi Z, Miller RO, Charlton-Ouw CC 3, Codreanu KM, Saqib ME, Safi NU, H. J., Estrera AL. Preoperative sarcopenia portends worse outcomes after descending thoracic aortic aneurysm repair. Ann Thorac Surg. 2018;106(5):1333–9. 10.1016/j.athoracsur.2018.05.060. 29944880 10.1016/j.athoracsur.2018.05.060

[25] Tanaka S, Ozeki N, Mizuno Y, Nakajima H, Hattori K, Inoue T, Nagaya M, Fukui T, Nakamura S, Goto M, Sugiyama T, Nishida Y, Chen-Yoshikawa TF. Preoperative paraspinous muscle sarcopenia and physical performance as prognostic indicators in non-small-cell lung cancer. J Cachexia Sarcopenia Muscle. 2021;12(3):646–56. 10.1002/jcsm.12691. 33665984 10.1002/jcsm.12691 PMC8200441

[26] Liu P, Hao Q, Hai S, Wang H, Cao L, Dong B. Sarcopenia as a predictor of all-cause mortality among community-dwelling older people: A systematic review and meta-analysis. Maturitas. 2017;103:16–22. 10.1016/j.maturitas.2017.04.007. 28778327 10.1016/j.maturitas.2017.04.007

[27] -Mosk CA, van Vugt JLA, de Jonge H, Witjes CDM, Buettner S, Ijzermans JNM, van der Laan L. (2018) Low skeletal muscle mass as a risk factor for postoperative delirium in elderly patients undergoing colorectal cancer surgery. Clin Interv Aging. 2018;13:2097–2106 10.2147/CIA.S175945 10.2147/CIA.S175945 PMC6205536 30425464

[28] Cooper C, Fielding R, Visser M, et al. Tools in the assessment of sarcopenia. Calcif Tissue Int. 2013;93:201–10. 10.1007/s00223-013-9757-z. 23842964 10.1007/s00223-013-9757-z PMC3744387

[29] Derstine BA, Holcombe SA, Ross BE, Wang NC, Su GL, Wang SC. Skeletal muscle cutoff values for sarcopenia diagnosis using T10 to L5 measurements in a healthy US population. Sci Rep. 2018;8(1): 11369. 10.1038/s41598-018-29825-5. 30054580 10.1038/s41598-018-29825-5 PMC6063941

[30] -Gomez-Perez S, McKeever L, Sheean P. Tutorial: A step-by-step guide (version 2.0) for measuring abdominal circumference and skeletal muscle from a single cross-sectional computed-tomography image using the National institutes of health ImageJ. JPEN. 2020;44:419–24. 10.1002/jpen.1721 31617218

[31] Bufano A, Cartocci A, Benenati N, Ciuoli C, Simon Batzibal M, Bombardieri A, Iraci Sareri G, Sannino I, Tirone A, Voglino C, Vuolo G, Castagna MG. New specific skeletal muscle mass index cut-offs for the assessment of sarcopenia in patients with severe obesity. Front Endocrinol (Lausanne). 2024;15: 1369584. 10.3389/fendo.2024.1369584. 39036048 10.3389/fendo.2024.1369584 PMC11258000

[32] Cruz-Jentoft AJ, Bahat G, Bauer J, Boirie Y, Bruyère O, Cederholm T, Cooper C, Landi F, Rolland Y, Sayer AA, Schneider SM, Sieber CC, Topinkova E, Vandewoude M, Visser M, Zamboni M, the Extended Group for EWGSOP2, Writing Group for the European Working Group on Sarcopenia in Older People 2 (EWGSOP2. Sarcopenia: revised European consensus on definition and diagnosis. Age Ageing. 2019;48(1):16–31. 10.1093/ageing/afy169. 30312372 10.1093/ageing/afy169 PMC6322506

